# What Sun Protection Practices Should Be Adopted by Trainee Teachers to Reduce the Risk of Skin Cancer and Other Adverse Outcomes?

**DOI:** 10.3390/ijerph18020529

**Published:** 2021-01-10

**Authors:** Guillermo De Castro-Maqueda, Carolina Lagares Franco, José V. Gutiérrez-Manzanedo, Fabriziomaria Gobba, Nuria Blázquez Sánchez, Magdalena De Troya-Martin

**Affiliations:** 1 Department of Physical Education, Faculty of Education Sciences and University of Cádiz, 11519 Cádiz, Spain; guillermoramon.decastro@uca.es; 2 Department of Statistics and Operational Research, University of Cádiz, 11510 Cádiz, Spain; carolina.lagares@uca.es; 3 Department of Biomedical, Metabolic and Neural Sciences, University of Modena and Reggio Emilia, 41121 Modena, Italy; fabriziomaria.gobba@unimore.it; 4 Dermatology Department, Costa del Sol Hospital, 29603 Marbella, Spain; nuriaderm1@gmail.com (N.B.S.); magdalenadetroya@gmail.com (M.D.T.-M.)

**Keywords:** skin cancer, photoprotection habits, photoexposure, university students, health sciences

## Abstract

Excessive sun exposure and insufficient protection are the main risk factors for the onset of melanoma and non-melanoma skin cancer (the most common types of cancer suffered by fair-skinned populations) and other adverse effects on the skin and eyes. Epidemiological data highlight the scant awareness of this risk among young people and the high rates of sunburn often recorded among this population. The main aim of the present study is to examine sun exposure and protection behavior by university students. A cross-sectional questionnaire-based study was undertaken to investigate sun exposure and protection practices among students of education sciences at a university in southern Spain. The data obtained were used to perform a descriptive, comparative analysis, by groups and by gender, of photoprotection and skin self-examination practices. The reliability and validity of the questionnaire were both tested. Of the 315 students who completed the questionnaire, 74.6% had suffered at least one sunburn during the previous year. Few made frequent use of sunscreen or protective clothing and 89.5% did not self-examine their skin. The metric properties of the questionnaire revealed its excellent reliability and validity. Among the Spanish university students considered, there was little awareness of the risk of excessive sun exposure, self-protection was insufficient, the potential exposure to dangerous levels of ultraviolet radiation was high, and most had suffered one or more sunburns in the last year. Intervention strategies should be implemented to highlight the risks involved and the need for more appropriate sun protection practices. Information campaigns should be conducted in this respect so that, when these students become teachers, they will have adequate knowledge of the risks involved and of the benefits of addressing this problem effectively, and will ultimately transfer these health education competences to their own students.

## 1. Introduction

Excessive exposure to solar radiation can impact on health in various ways, mostly related to the ultraviolet (UV) component of sunlight. Both acute and chronic effects may be induced, damaging the skin and the eyes in particular [1]. Solar radiation and UV light are both classified as Group 1 carcinogens by the International Agency for Research on Cancer [2], and can induce basal cell carcinoma (BCC) and squamous cell carcinoma (SCC), the most common cancers in fair-skinned populations worldwide [3]. Malignant melanoma is also correlated with solar UV radiation (UVR). Furthermore, skin cancer is a frequent, even if underreported, occupational disease in outdoor workers [4]. Over the last 30 years, the prevalence of skin cancer has risen continuously in Spain and elsewhere [5,6], but UVR exposure is also related to other chronic skin and eye disorders such as photo-aging, actinic keratosis, pterygium, cataracts, and possibly macular degeneration, another disease whose incidence has increased significantly [1,7].

UVR is a major cause of skin cancer, provoking high rates of both melanoma and non-melanoma skin cancer. In Europe, the incidence of these skin cancers has increased progressively, from 3–8% per year, since 1960. In 2017, for example, 7.7 million cases of non-melanoma skin cancer were diagnosed in Europe [8]. Survival rates vary from 90% in western European countries to less than 60% in the east [3].

The highest rates of melanoma skin cancer are recorded in Norway and Switzerland, with an average of 20 new cases per 100,000 inhabitants per year. In Spain, the incidence is around 6–8 cases per 100,000 inhabitants per year. The highest rates of non-melanoma skin cancer are recorded in Ireland and Switzerland, with an average of 140 cases per 100,000 inhabitants per year. In Spain, the incidence is around 110 cases per 100,000 inhabitants per year.

Biological effects on humans, such as sunburn (erythema), DNA damage, or vitamin D skin-synthesis depend on the UV wavelength, which ranges from 250–400 mm. The UV Index (UVI) range from zero upward, describes the level of solar UV radiation at the Earth’s surface. UVI 6 to 7 is defined as high, UVI 8 to 10 is considered very high, and UVI 11+ is defined as extreme [9]. The higher the value of UVI, the shorter the sunburn time. In Spain, one of the sunniest countries in Europe, high or very high values of UVI are recorded during 40% of the year. During June, July, and August, the average maximum UVI is 8–9 [10].

UV-induced adverse effects are related to a photochemical mechanism that depends on the total dose, which is given by the product of the duration of the exposure and the intensity of the radiation, known as the principle of reciprocity, or the Bunsen–Roscoe law. The effect produced is cumulative throughout life, and resultant disease may appear several years after the first exposure, when it is too late to take preventive countermeasures [11]. Furthermore, the amount of exposure received during the first 20 years of life has a decisive influence on the risk of developing skin cancer in later life. In this respect, sunburn is a critical risk factor: a single episode of sunburn in childhood or adolescence doubles the risk of melanoma [12,13].

Most of these effects, if not all, can be prevented by proper sun-safety education, including knowledge of the differences in sun-sensitivity according to the skin phototype [14], fostering the adoption of more effective sun protection practices, such as avoiding exposure at times of maximum incidence of UVR, making use of available shade, wearing hats, sunglasses and appropriate clothing, and regularly applying sunscreen cream with a protection factor of 30 or more [12,13,15].

Patterns of behavior concerning sun exposure and protection are usually evaluated by means of questionnaires, but the measurement properties of these instruments (for example, their validity, reliability, and sensitivity to change) should be properly tested and confirmed before use [16,17,18].

With respect to the relationship between sun exposure and protection and the development of skin cancer, there exist very few standardized instruments with which to study these practices [19,20,21], and the questionnaires that have been used in previous research present great variability in their design and content. Moreover, in many cases they have not been previously validated.

Epidemiological studies, conducted elsewhere in Spain and also abroad, have confirmed the existence of high rates of sunburn among university students, regardless of their skin type [14] and a vulnerability related to risky behavior regarding sun exposure and protection [22,23,24,25,26,27]. These findings highlight the need to consider university students as a high-risk group for skin cancer and to design specific prevention strategies for this target group [21]. Interestingly, there is a growing evidence that occupational sun-safety education can be effective in increasing workers’ sun-protection habits [28].

The main aim of the present study is to examine the sun exposure and protection behavior of students of education sciences at a university in southern Spain.

## 2. Materials & Methods

### 2.1. Study Design and Scope

This cross-sectional observational study analyzes the responses made by university students of Education Sciences to a questionnaire on their sun exposure and protection practices. The study was carried out during October and November 2019, based on a convenience sample recruited at the University of Cádiz (Spain).

### 2.2. Participants and Selection Criteria

The survey was conducted on a sample of 315 university students, aged 18–46 years, grouped as follows, according to the faculty in which they were enrolled: GI (pre-school education), GII (primary education), and GIII (physical education).

The following inclusion criteria were applied: students aged 18 years or older, enrolled in one of the faculties of Education Sciences or Physical Activity at the University of Cádiz, with adequate understanding of spoken and written Spanish, who voluntarily agreed to participate in this project, and provided signed informed consent.

### 2.3. Method

Participants were recruited at the beginning of the academic year, in October 2019, and invited to complete a self-administered two-page questionnaire about sun exposure and protection practices. The questionnaire was designed to be completed in 5–10 min, and one of the research team was present at all times to resolve any questions that might arise.

To study the metric properties of the questionnaire, the questionnaire was returned to the same students two weeks after the first data collection and re-assessed. Participation in the study was completely voluntary. The participants’ anonymity was protected by assigning a code to each student. 

### 2.4. Questionnaire

A self-administered two-page questionnaire on individual sun exposure and protection practices, previously validated and published by Glanz et al. [18], was completed by each participant. It was translated from English into Spanish by a group of experts, by consensus on the content of the items translated, and adapted for use with university students. In addition, the questionnaire was expanded with some further items recommended in the literature and considered relevant for this research [29,30,31].

In addition the items included in the questionnaire, the following information was obtained for each respondent: sociodemographic variables (age, sex, university subject studied, and family history of skin cancer); sun exposure practices (number of hours with skin exposed to the sun, between 10 a.m. and 4 p.m., during the week and at weekends); number of sunburn episodes in the last year (defined as pain and redness of the skin lasting more than one day); sun protection measures when exposed to the sun, such as making use of shade, wearing sunglasses, a cap or hat, a long-sleeved t-shirt, and long trousers; type of skin (Fitzpatrick phototype) [14]; use of sunscreen, its protection factor and rate of reapplication; regular personal skin examination and visits to a dermatologist; diagnosis, if any, of skin cancer; outdoor physical activity (hours per day); during physical activity, use of sunscreen, its protection factor, and rate of reapplication.

The main study variables on sun exposure and protection were presented in the questionnaire as follows: 

#### 2.4.1. Sun Exposure Practices

##### Part 1

*Sun exposure and protection practices*: (a) Average time spent in the sun between 10 a.m. and 4 p.m. on weekdays refers to summer exposure (Item 1). (b) Average time spent in the sun at the weekend (Item 2). In both cases, responses were made according to a 6-point Likert scale (0 = less than 30 min; 1 = between 31 and 60 min; 2 = two hours; 3 = three hours; 4 = four hours; 5 = five hours; and 6 = six hours). 

*Sunburn:* events experienced during the previous year, on a range from 0 to “5 or more”. Sunburn is defined as the presence of blistering and/or reddening and/or pain lasting more than one day (Item 3).

*Sun protection practices*: as recommended by the World Health Organization: using sunscreen (Item 4), a long-sleeved t-shirt (Item 5), and a cap or hat (Item 6), staying in the shade (Item 7), and wearing sunglasses (Item 8). As a risk practice, participants were asked about sun exposure performed in order to acquire a tan (Item 9). All responses were recorded on a 4-point Likert scale (1 = Rarely or never; 2 = Sometimes; 3 = Often; 4 = Always).

*Skin color*: Color of skin not exposed to sunlight (Item 10), (six response categories: pale; fair; intermediate; moderate brown; dark brown; darkest brown; corresponding to those of the Fitzpatrick phototype) [15].

##### Part 2 

*Skin check-up:* The students were asked if they had ever had a medical check-up of their skin (yes/no) (Item 11), and if so, when the last time had been (month and year), (Item 12). They were also asked if they themselves or someone else had examined their skin, including their back, in the past year, to search for spots or lesions (Item 13). If the answer was affirmative, the number of times such an examination had been made was also recorded. (Item 14).

##### Part 3

*Physical activity*. The following questions were prepared by a group of experts in physical education and sports, and included in the questionnaire.

- On average, how many hours of outdoor physical activity do you perform each day? (Item 15).
- When you are performing outdoor physical activity, do you usually put sunscreen on your face? (Item 16). If so, what sun protection factor does the sunscreen have? (Item 17).
- Do you usually reapply sunscreen throughout the day, how often? (Item 18).

### 2.5. Statistical Analysis

A sociodemographic and descriptive analysis was made of all the information obtained from the study sample, using frequency tables for the qualitative data and descriptive statistics for quantitative data (mean, standard deviation (SD), maximum, and minimum).

Chi-square tests were performed to calculate the association between the qualitative variables, assuming a level of significance of 5%. The *p*-value was interpreted as the probability of there being no differences between the groups. 

The metric properties (i.e., the validity and reliability) of the questionnaire were tested by principal components analysis (PCA) with varimax rotation and eigenvalues greater than one. The validity of the construct was assessed by the factor saturations of the rotated component matrix. The validity of the PCA was evaluated by the Kaiser–Meyer–Olkin (KMO) test and by Bartlett’s test of sphericity, and the degree of variance explained was recorded. According to the qualitative or quantitative nature of the items considered, the intraclass correlation coefficient (ICC) and the Kappa coefficient were calculated to determine the reliability-stability of two evaluations performed. Internal consistency, also expressed in terms of reliability, was evaluated by Cronbach’s alpha coefficient. All analyses were performed using IBM SPSS v.22 statistical software (IBM Company, New York, NY, USA).

## 3. Ethical Considerations

This study was approved by the Research Ethics Committee of the University of Cádiz in March 2018 and Costa del Sol Hospital (n85-05-2019). The study was conducted in full accordance with the Helsinki Declaration and with Spanish legislation on patient confidentiality (Law 41/2002). All data were recorded and stored anonymously, in strict accordance with the currently applicable laws and regulations on data protection and digital rights (EU Regulation Data Protection, 2016/679; Organic Law 3/2018, of 5 December).

## 4. Results

The questionnaire was completed by 315 university students from the Faculty of Education Sciences at the University of Cádiz (Spain). By area of specialization, they were distributed as follows: pre-school education (39.7%), primary education (13.7%), and physical education/secondary education (46.6%). The study groups presented significant differences by sex (*p* < 1.93·10^−21^) and by age (*p* = 0.014). Just over half of the sample (52.2%) were male. The students’ average age was 21.23 years (SD: 3.2). The youngest was 18 years old and the oldest was 46. In response to the question about a family history of skin cancer, 96% of the responses were negative.

### 4.1. Descriptive Results

Table 1 shows the descriptive results obtained for the students’ photoexposure practices. Significantly, 40.2% spent three hours or more exposed to the sun between 10 a.m. and 4 p.m. from Monday to Friday (Item 1) and this proportion increased to 57.5% over the weekend (Item 2). 74.6% had experienced at least one sunburn event in the last twelve months (Item 3) and 40% had fair or very fair (pale) skin (Item 10). The tests of dependence among the study variables revealed significant differences by gender for Item 10 (*p* < 0.011).

As shown in Table 1, 67.8% of these students were exposed to the midday sun for at least two hours on weekdays, a proportion that rose to 78.9% at the weekend. This level of exposure means that many students received a high level of sun exposure during the year, and were at considerable risk of sunburn if they were not adequately protected. Indeed, in the previous summer 74.6% had experienced at least one painful sunburn.

With respect to sun protection, Table 2 shows that almost half of the respondents either did not use sunscreen or did so only occasionally. In addition, most of these students rarely or never wore a cap or hat. In items 4, 5, and 9, there were statistically significant differences by gender, with women significantly more likely to respond “often” and “always” to Items 4 and 9 (*p* < 0.003 and *p* < 4.88·10^−9^, respectively) and men more likely to wear a long-sleeved t-shirt (Item 5) (*p* < 1.69·10^−11^). Perhaps the most significant finding reflected in this table is the proportion of regular use (“often/always”) of the following sunscreen measures: thus, sunscreen 53.3%, long-sleeved t-shirt 36.6%, cap or hat 10.2%, shade 50.1%, and sunglasses 46.7%. This pattern of sun protection is clearly inadequate, and explains the high rates of sunburn reported by the students.

Table 3 shows the descriptive results obtained for Parts 2 and 3 of the questionnaire. With respect to self-examination of the skin, Item 12 is not shown, as the information obtained refers to the date, and only applies if an affirmative answer is given to Item 11.

According to the responses made to Item 14 (number of times the student had examined his/her own skin in the last year) - only made if an affirmative answer was given to Item 13- on average, the students had examined their skin 3.2 times in the last year (SD: 2.6). The minimum value stated was 1 and the maximum, 10.

A significant finding revealed in Table 3 is that only 5.4% of the students had had a medical examination of the skin, and only 10.5% had examined their own skin.

According to the students’ responses to Part 3 of the questionnaire, on sun protection practices during physical activity outdoors, the average time spent in this respect (Item 15, not shown in the table) was 1.84 h per day (SD: 1.5), ranging from 0 to 10 h. Only 17.9% of the students used sunscreen on the face, the area of skin that is most exposed to UVR. This inadequate protection is aggravated by the fact that only 10.2% of the students habitually wear a cap or hat as protection against the sun.

Male and female students did not vary significantly in their attitudes towards skin check-ups, but differences in protection practices were observed. Although the majority of students did not use sunscreen for the face, more women than men did so (67% vs. 33%; *p* < 0.002). Similarly, women were more likely to reapply the cream (63% vs. 37%; *p* < 1.6·10^−5^).

Although 76.8% of those who applied sunscreen used one with an adequate protection factor (≥30), only 49.3% reapplied it within two hours. Failure to reapply the necessary protection compromises its effectiveness in preventing sunburn.

### 4.2. Metric Properties 

Table 4 shows the results obtained from the principal component analysis. The KMO test confirmed the adequacy of this analysis (0.577) and the Bartlett sphericity test confirmed the relevance of the factor model (*p* < 3.04·10^−72^). Four factors that accounted for 63.06% of the variance were extracted: the first of these, hours of sun exposure, was strongly correlated with Items 1 and 2. Factor 2, avoid sun exposure, was positively correlated with Items 5 and 7 (wearing protective clothing; staying in the shade) and negatively correlated with Item 9 (sunbathing in order to tan). Factor 3, protective measures, correlated with Items 4, 6, and 8 (sunscreen, headgear, sunglasses), while Factor 4, sunburn and type of skin, was positively correlated with Item 3 (sunburn history) and negatively with Item 10 (skin color).

PCA with varimax rotation was performed on the items with proven reliability, i.e., absolute agreement according to Cohen’s Kappa coefficient (Items 11, 13, 16, 17, and 18). Although from the formal standpoint of PCA, the variables should be quantitative, this approach can also be used with dichotomous variables (as is the present case) to determine patterns of relationships between the variables. In this analysis, we obtained two main components that accounted for 53.89% of the variability of the data. The KMO test confirmed the adequacy of the factor analysis (0.528) and Bartlett’s test of sphericity confirmed that of the factor model (*p* < 9.94·10^−14^). The first component correlated positively with Items 11 (professional skin check-up) and 13 (personal skin check-up), with correlations of 0.854 and 0.826, respectively; the second component correlated with Items 16 (protection during outdoor activity), 17 (sun protection factor > 30), and 18 (reapplication of sunscreen), with correlations of 0.48, 0.747, and 0.684, respectively. These results are coherent with the questionnaire construct.

Table 5 shows the results obtained from our evaluation of the reliability of the instrument using the ICC or the Kappa coefficient concerning absolute agreement and the Cronbach alpha coefficient for internal consistency. In general, good results were obtained, thus confirming the reliability of the instrument.

## 5. Discussion

Sunburn, especially at a young age, is considered a main risk factor for malignant melanoma, the most aggressive form of skin cancer, and also for non-melanoma forms such as BCC, the most common type. Our findings show that university students are alarmingly exposed to sunburn, with nearly 75% experiencing such a lesion during the previous year. This rate is similar to that found in the USA, where 76% of university athletes had had 1–3 sunburns in the previous year [32,33], and in Brazil, where 67% of university students had suffered this experience [34]. Nevertheless, even higher rates have been observed in other contexts, for example, the 87% reported for nursing students in Spain [22].

In this study, we analyzed the sun exposure and protection practices of Spanish university students, using an adaptation of the questionnaire on the prevention of skin cancer in adults proposed by Glanz et al. [34]. When applied to university students, this questionnaire has good metric properties in terms of reliability and validity. Questionnaires provide an effective means of measurement in population studies and are commonly used in health research. [23].

Adverse UVR effects on the skin depend not only on the duration of exposure, but also, and to a larger degree, on the skin type. Thus, Fitzpatrick phototypes 1 and 2, representing very fair skin, are the most sensitive to UV damage, both acute and long term [14]. In general, there are no differences between the sexes in this respect, although women are more likely than men to take protective measures such as making frequent use of sunscreen and wearing suitable headgear [35].

In the present study, the participants were university students, taking degrees in aspects of education science. Analysis of the findings obtained confirmed our initial hypothesis that, possibly due to their youth, these students took insufficient measures of sun protection and were at serious risk of developing skin lesions and cancer.

The University of Cádiz is located in southwest Spain, where levels of UVR are high throughout the year. In some respects, the sun is beneficial to healthy living, for example in contributing to the supply of vitamin D, as indicated by studies carried out with nurses [36,37]. However, prolonged exposure to the sun presents a tangible risk to health [38]. According to our study, 60% of the students considered are exposed to UVR for 1–3 h between 10 a.m. and 4 p.m. during the week, and this figure rises to 63% at the weekend. This degree of solar exposure is intensive and potentially harmful.

To our knowledge, no previous studies of the sun exposure and protection practices of university students have focused on the area of education sciences; our review of the literature shows that most have analyzed students of health sciences, other areas of science, or the humanities [22,25,26,27,33,39], while an Italian study focused on high school students and outdoor workers in the agriculture and construction sectors [40]. The overall conclusions drawn in almost all of these studies are coherent with our own findings that the participants’ sun exposure and protection practices are often inappropriate.

A notable finding of the present study is that well over half of the respondents (59.7%) had suffered one or more sunburns during the previous year, following sun exposure in order to get a tan. This result corroborates earlier research conducted in this area [41].

In general, these students make inadequate use of means of sun protection. Thus, 46.7% do not use sunscreen at all, or do so only rarely. This result is alarming and highlights the need to promote the use of sunscreen (moreover, emphasizing the need to reapply it regularly, especially after contact with water or following exertion resulting in a significant degree of perspiration) [42]. When outdoor physical activity is performed, the situation is even more critical; in this situation, 82.1% of the participants in our study do not use sunscreen on the face.

The inadequate use of sunscreen is accompanied by problems in other areas, too. Thus, 73.8% of the students in our analysis never or only rarely wear a hat or cap to protect their skin from the sun. Indeed, this practice is the least commonly adopted by the respondents. Previous studies have reported similar findings [37,42,43], observing also that, within this minority of users, men are more likely than women to wear protective headgear.

Another important deficiency concerns the insufficient use of sunglasses, which provide an important degree of protection against UV rays and can even prevent injuries such as cataracts. Over half of the participants in this study (53.7%) rarely or never wear sunglasses, although the women do so more frequently than the men. These results corroborate previous reports [40,43] and are significant for two reasons: because of the cancer risk to the persons directly involved, and because in the near future these men and women will be teaching children and adolescents and should provide them with a positive role model, exemplifying good practices. In this respect, studies have shown that many primary and secondary school teachers in Spain do not protect themselves properly from UVR [32]. We believe it necessary to raise awareness of the seriousness of the present situation and of its possible consequences for future generations. Therefore, these students of education sciences need to be informed of the dangers they face and urged to improve their sun protection practices.

Focusing on the proportion of respondents who visit a dermatologist, the numbers are shocking. Thus, 94.6% have never had their skin examined for spots or lesions, although this simple act is one of the most effective measures they can take to detect skin lesions caused by exposure to UVR [44].

Another worrying finding is that most of our respondents (89.5%) do not perform a self-examination of their skin to detect sun-related injuries. This suggests there is a low level of awareness of the personal risk of skin cancer. The failure to examine one’s own skin, or to have this done by a dermatologist, may result in a delayed diagnosis of cancer and possibly a poorer prognosis. This aspect of our study corroborates previous research conducted with young athletes [29,30].

Test-retest reliability was confirmed by the Kappa coefficients and the ICC obtained. The Cronbach’s alpha coefficient obtained also reflected good internal consistency, with values ranging from 0.374 to 0.65 [45]. These values are not especially high, which is a good sign, as a high coefficient may denote redundancy in the information collected. The lowest values were obtained for Items 16, 17, and 18 (related to physical activity and sun protection), which are dichotomous and therefore only require us to calculate the Kuder–Richardson KR-20 coefficient [46]. The values obtained suggest there may be unrelated subconstructs [47].

The factor analysis performed revealed four underlying dimensions in the information compiled: duration of sun exposure (factor 1); avoiding sun exposure (factor 2); effective protection (factor 3); and lesions caused by sun exposure (factor 4).

The results obtained in this study show that the university students surveyed do not protect their skin effectively from UVR. The use of sunscreen alone is not sufficient to protect the skin; further actions must be taken, such as making use of available shade, wearing sunglasses and suitable headgear, and wearing a long-sleeved shirt and long trousers. Accordingly, efforts should be made to promote changes in students’ attitudes towards sun exposure and protection [48]. It has been shown that focused interventions can increase the use of sunscreen, and that the image presented by the teacher is of crucial importance in transferring a good understanding of these issues to subsequent generations of students, thus enhancing their sun protection practices [49]. Finally, there is growing evidence that safety education can be effective in improving sun protection habits [28,50].

## 6. Strengths and Limitations

The main strength of the present study resides in the solid theoretical foundations of the questionnaire used, which is based on the recommendations of experts in the field such as Glanz et al. In consequence, the results obtained provide good internal consistency and stability. The data presented are significant in two respects: on the one hand, we highlight the risk of skin cancer currently faced by these university students. On the other, these same students will in the near future become teachers themselves, responsible for educating young people in these and other matters.

To our knowledge, this is the first study of its type to be conducted with a population consisting of Spanish university students of Education Sciences, who will subsequently be teachers in three areas of education in Spain—pre-school, primary, and secondary. If these students are targeted today with campaigns informing them about sun exposure and effective measures of protection, this will greatly contribute to their transmitting this information to future generations of students, aged from 3 to 18 years.

Our study also has certain limitations. Firstly, the use of a questionnaire to obtain the study data means that there is no objective measurement of the variables considered, such as the sun protection measures taken. Nevertheless, Glanz et al. [18] observed a good correlation between self-reported data and measurements obtained of UV exposure. Another weakness of the present research is that the questionnaire used was not subjected to a strict process of validation.

In future research, it would be interesting to evaluate results by skin color/tanning susceptibility, as these factors may also influence young people’s sun exposure and protection practices.

## 7. Conclusions

In conclusion, the results of our study indicate that Spanish university students are potentially exposed to dangerous levels of UV radiation and that many do not adopt adequate sun protection habits, despite having suffered one or more sunburns during the previous year. Therefore, intervention strategies should be designed and applied to highlight the risks involved, to improve sun protection practices and to encourage skin examination. In turn, this will raise awareness of the relationship between excessive exposure to the sun and the development of skin cancer.

This paper reports the currently excessive levels of sun exposure and the inadequate sun protection practices observed among Spanish university students of Education Sciences. Information campaigns should be conducted to address this problem so that when these students become teachers and have students of their own they may describe and demonstrate the benefits of skin care and provide a good foundation in health education. In presenting such campaigns, care should be taken to balance the message, addressing both the health risks of sun exposure and the possible benefits, such as the enhanced synthesis of vitamin D. 

Therefore, to reduce the likelihood of sunburn and other damage to the body and eyes from prolonged exposure to the sun, the following measures are recommended.

- Minimize exposure to the sun during peak-sun hours (10 a.m. to 3 p.m.).
- Avoid prolonged sun exposures. In general, it takes as little as 20 min a day to maintain an adequate level of vitamin D.
- Apply sunscreen with SPF15 or higher to all exposed areas of the body.
- Repeat the application of sunscreen every two hours, even on cloudy days.
- Wear clothes that fully cover the body.
- Avoid unnecessary exposure to UV radiation with lamps or tanning beds.
- Protect children from excessive exposure to the sun during peak-sun hours and apply sunscreen to children aged over 6 months.
- Wear sunglasses outdoors on sunny days. The regular use of sunglasses also offers some protection against UV rays.
- Wear a wide-brimmed hat to protect the eyes (and if possible, a hat shading the back of the neck, to protect the skin).

## Figures and Tables

**Table 1:** Sun exposure practices, sunburn events, and skin type.

Time	Weekday Exposure (Item 1) *n* (%)	Weekend Exposure (Item 2) *n* (%)	Sunburns	Sunburn Events Last Year (Item 3) *n* (%)	Colour	Skin Colour (Item 10) *n* (%)
0–60 min	101 (32.2)	66 (21.1)	None	80 (25.4)	Pale	15 (4.8)
120 min	87 (27.7)	67 (21.4)	One	115 (36.5)	Fair	116 (36.8)
180 min	66 (21.0)	67 (21.4)	Two	73 (23.2)	Intermediate	49 (15.6)
240 min	31 (9.9)	68 (21.7)	Three	31 (9.8)	Moderate brown	99 (31.4)
300 min	19 (6.1)	19 (6.1)	Four	6 (1.9)	Dark brown	31 (9.8)
360 min	10 (3.2)	26 (8.3)	Five or more	10 (3.2)	Darkest brown	5 (1.6)

**Table 2:** Sun protection practices and tanning preferences.

Items	Seldom or Never *n* (%)	Sometimes *n* (%)	Often *n* (%)	Always *n* (%)
Item 4. Use sunscreen	58 (18.4)	89 (28.3)	85 (27.0)	83 (26.3)
Item 5. Wear long-sleeved t-shirt	132 (42.0)	67 (21.4)	71 (22.6)	44 (14.0)
Item 6. Wear cap or hat	231 (73.8)	50 (16.0)	20 (6.4)	12 (3.8)
Item 7. Stay in the shade	41 (13.1)	115 (36.7)	105 (33.5)	52 (16.6)
Item 8. Wear sunglasses	89 (28.3)	79 (25.1)	91 (28.9)	56 (17.8)
Item 9. Sunbathe in order to tan	74 (23.5)	69 (22.0)	88 (28.0)	83 (26.4)

**Table 3:** Skin check-up and sun protection during outdoor activity.

Skin Check-Up			*n*	%
Part 2	Item 11. Medical examination	No	297	94.6
		Yes	17	5.4
	Item 14. Self-examination of skin	No	280	89.5
		Yes	33	10.5
Sun protection during outdoor activity				
Part 3	Item 16. Sunscreen on face	No	253	82.1
		Yes	55	17.9
	Item 17. Factor ≥ 30	No	69	23.2
		Yes	229	76.8
	Item 18. Reapplication	No	151	50.7
		Yes	147	49.3

**Table 4:** Correlations between questionnaire items and factors.

ltems	Factor 1	Factor 2	Factor 3	Factor 4
ITEM 1 Weekday exposure	0.906	−0.066	−0.033	0.035
ITEM 2 Weekend exposure	0.896	−0.128	0.035	−0.001
ITEM 3 Sunburn history	0.172	0.019	−0.209	0.839
ITEM 4 Use sunscreen	−0.111	−0.157	0.712	−0.142
ITEM 5 Wear long-sleeved t-shirt	−0.057	0.751	−0.060	0.091
ITEM 6 Wear cap or hat	−0.051	0.135	0.652	0.054
ITEM 7 Stay in shade	−0.173	0.655	0.273	−0.153
ITEM 8 Wear sunglasses	0.174	0.138	0.572	0.040
ITEM 9 Sunbathe in order to tan	0.006	−0.821	−0.017	0.044
ITEM 10 Color of skin	0.245	0.120	−0.422	−0.653

**Table 5:** Reliability with respect to stability (intraclass correlation coefficient (ICC) and Kappa) and internal consistency (Cronbach’s alpha).

Item	ICC	Cronbach’s Alpha
1, 2 (Sun exposure)	0.88	0.897
3 (Sunburns)	0.91	0.906
4–9 (Sun protection)	0.52	0.560
10 (Skin color)	0.97	0.974
12 (Last medical visit)	0.96	0.962
14 (Skin self-exam)	0.73	0.735
15 (Physical activity)	0.90	0.91
Item	Kappa	S.E.
11 (Medical exam)	0.65	0.095
13 (Self-exam)	0.75	0.064
16 (Sunscreen face)	0.68	0.055
17 (SPF > 30)	0.68	0.051
18 (Reapplication)	0.67	0.043

SPF means sun protection factor.
